# Copy number variations in ultrasonically abnormal late pregnancy fetuses with normal karyotypes

**DOI:** 10.1038/s41598-020-72157-6

**Published:** 2020-09-15

**Authors:** Meiying Cai, Na Lin, Linjuan Su, Xiaoqing Wu, Xiaorui Xie, Ying Li, Yuan Lin, Liangpu Xu, Hailong Huang

**Affiliations:** Department of the Prenatal Diagnosis Center, Fujian Maternity and Child Health Hospital, Affiliated Hospital of Fujian Medical University, Fujian Key Laboratory for Prenatal Diagnosis and Birth Defect, Fuzhou, China

**Keywords:** Medical genetics, Molecular medicine

## Abstract

Many fetuses are found to have ultrasonic abnormalities in the late pregnancy. The association of fetal ultrasound abnormalities in late pregnancy with copy number variations (CNVs) is unclear. We attempted to explore the relationship between types of ultrasonically abnormal late pregnancy fetuses and CNVs. Fetuses (n = 713) with ultrasound-detected abnormalities in late pregnancy and normal karyotypes were analyzed. Of these, 237 showed fetal sonographic structural malformations and 476 showed fetal non-structural abnormalities. Single nucleotide polymorphism (SNP)-based chromosomal microarray (CMA) was performed on the Affymetrix CytoScan HD platform. Using the SNP array, abnormal CNVs were detected in 8.0% (57/713) of the cases, with pathogenic CNVs in 32 cases and variants of uncertain clinical significance (VUS) in 25 cases. The detection rate of abnormal CNVs in fetuses with sonographic structural malformations (12.7%, 30/237) was significantly higher (P = 0.001) than that in the fetuses with non-structural abnormalities (5.7%, 27/476). Overall, we observed that when fetal sonographic structural malformations or non-structural abnormalities occurred in the third trimester of pregnancy, the use of SNP analysis could improve the accuracy of prenatal diagnosis and reduce the rate of pregnancy termination.

## Introduction

Current techniques used for genetic testing of fetuses with ultrasound abnormalities include karyotype analysis and chromosomal microarray analysis (CMA). Karyotype analysis is presently the gold standard for prenatal diagnosis, but its resolution is low^1^. Thus, submicroscopic deletions or duplications (smaller than 3–5 Mb) may not be detected with traditional cytogenetic analysis unless additional techniques such as fluorescence in situ hybridization (FISH) are used. CMA is a high-resolution and genome-wide screening screening technology for the human genome. It is divided into two categories: Microarray based Comparative Genomic hybridization (aCGH) and single nucleotide polymorphism array (SNP), both of which can detect chromosomal microdeletions or microduplications. SNP array can detect not only CNVs, but also uniparental disomy and chimera. CMA has the advantage of high throughput and high resolution, and has been proven to be a powerful diagnostic tool in cases with developmental delays/mental retardation, autism syndrome, multiple birth defects, etc^2–4^. With the wide application of CMA as a prenatal diagnostic technique, clinical values of chromosomal microdeletions and microduplications are increasingly recognized.

Abnormal ultrasound findings in the second and third trimesters of pregnancy allow the detection of a unique group of late developmental manifestations, including major and minor fetal abnormalities or ultrasound soft markers, which do not exist in the first trimester of pregnancy^5^. Because of the lack of technical expertise in hospitals, obstetric ultrasound examination is only carried out in the second and third trimesters of pregnancy and some structural abnormalities are found only later with fetal development, thus resulting in a missed examination time for chorionic villus sampling and amniocentesis (Typically, chorionic villus sampling is performed at 10–12 weeks’ gestation, and amniocentesis is performed at 15–18 weeks’ gestation). Hence, at these later points, it becomes necessary to employ cordocentesis for cytogenetic analysis and to further clarify the cause of abnormality.

The relationships between fetal ultrasound abnormalities during pregnancy and chromosomal copy number variations (CNVs) have been extensively investigated^3,6,7^. In recent years, CMA has been recommended as the preferred diagnostic method for prenatal diagnosis of fetal ultrasound abnormalities^8^. However, there was little research assessment of the utility of CMA used in fetuses with abnormal ultrasound in late pregnancy. This study retrospectively analyzed the results of SNP analysis of 713 cases, from 2016 to 2019, and explored the relationship between types of ultrasonically abnormal late pregnancy fetuses and CNVs.

## Methods

### Patient data

**Figure 1:**
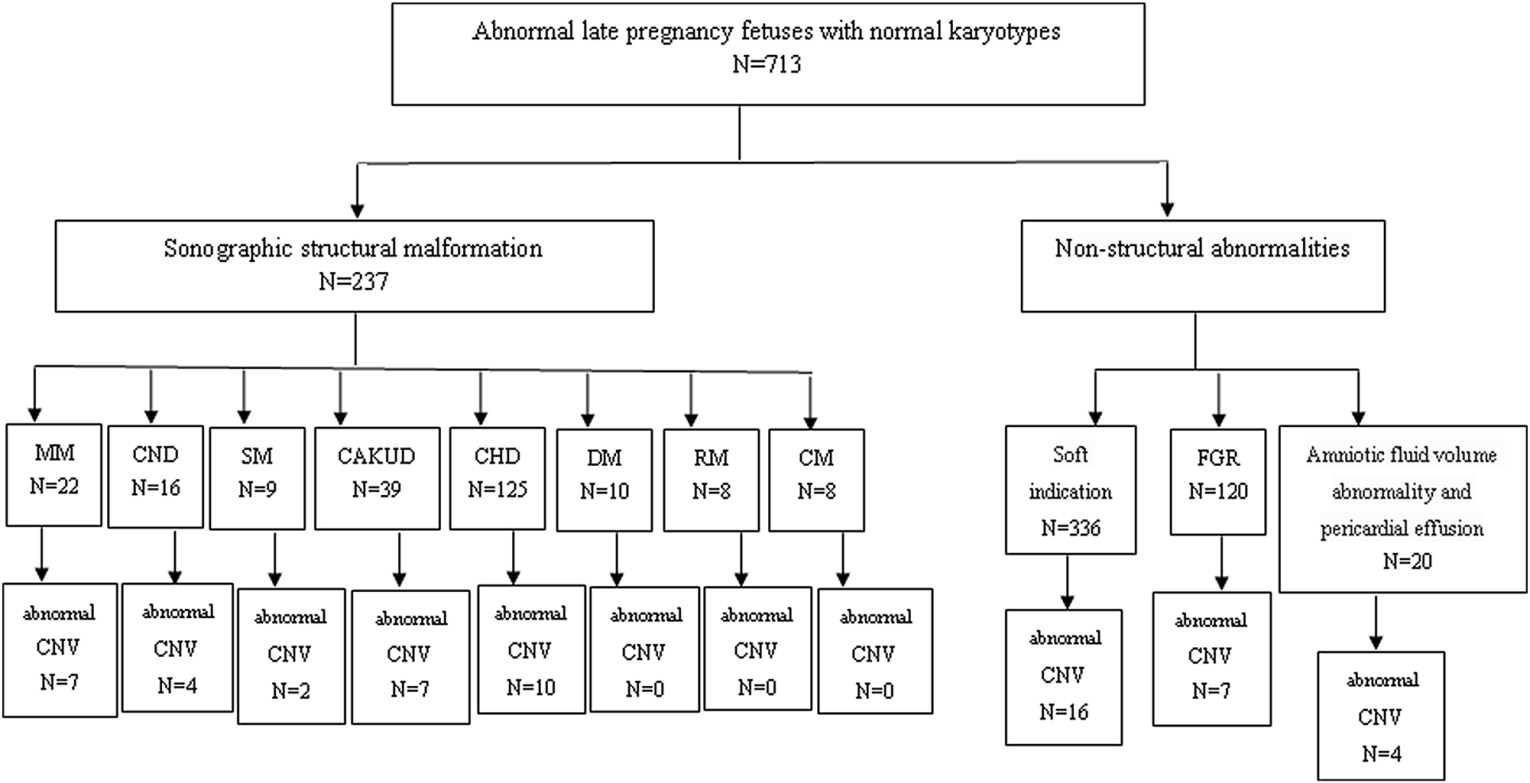
Ultrasonically abnormal late pregnancy fetuses with normal karyotypes were selected from November 2016 to July 2019. *MM* multiple malformations, *CND* central nervous disease, *SM* skeleton malformation, *CAKUD* congenital heart disease, *DM* digestive malformation, *RM* Respiratory malformation, *CM* craniofacial malformation, *FGR* fetal growth restrictions, *CNV* copy number variation.

The data regarding pregnancies with abnormal ultrasound findings (n = 713) from November 2016 to July 2019 were collected at the Fujian Maternal and Child Health Hospital. The exclusion criteria were abnormal karyotype analysis results and normal fetal ultrasound findings. The inclusion criteria were fetal sonographic structural malformations and non-structural abnormalities. According to the anatomical system affected, sonographic structural malformations were divided into: (1) central nervous system; (2) cardiovascular system; (3) urogenital system; (4) skeletal system; (5) respiratory system; (6) digestive system; and (7) craniofacial region malformations. Non-structural abnormalities were divided into: (1) ultrasound soft markers (including an echogenic bowel, absence of nasal bone, lateral ventricle widening, intracardiac echogenic focus, and tricuspid regurgitation); (2) fetal growth restriction (FGR); and (3) amniotic fluid volume abnormality and pericardial effusion. Indications for late cordocentesis included fetal sonographic structural malformations (n = 237) and fetal non-structural abnormalities (n = 476) (Fig. 1).

### Karyotype analysis

According to the routine methods established in our laboratory, umbilical cord blood was routinely cultured for 72 h, and then the chromosomes were harvested, fixed and prepared. Cultured cells were analyzed by karyotype analysis using Giemsa banding at a resolution of 450–550 bands.

### Single-nucleotide polymorphism (SNP) array

Ultrasound-guided cordocentesis was used to extract umbilical cord blood for prenatal SNP-array testing. To avoid maternal cell contamination during cordocentesis, short-tandem repeats analysis was conducted before testing fetal samples. Genomic DNA extraction from fetal umbilical cord blood cells was performed using a QIAamp DNA blood mini kit (Qiagen, Germany). The concentration and purity of genomic DNA were measured using a NanoDrop One Microvolume UV–Vis spectrophotometer (Thermo Fisher Scientific, USA). DNA digestion, amplification, purification, fragmentation, labeling, hybridization, washing, staining, scanning, and other steps were carried out according to the manual for an Affymetrix Genome CytoScan 750K gene chip. The reporting thresholds for CNVs were 400 kb deleted and 400 kb duplicated. Data analysis was carried out using the Chromosome Analysis Suite software 3.3 (Affymetrix, Santa Clara, CA, USA). Based on the nature of CNVs detected, CNVs were classified as pathogenic, variants of uncertain clinical significance (VUS), and benign, according to the American College of Medical Genetics guidelines^9^.

### Statistical analysis

IBM SPSS Statistics 20 (IBM, Armonk, NY, USA) was used for statistical analysis. The CNV rates were compared between fetuses with sonographic structural malformations and those with non-structural abnormalities. A value of P < 0.05 was considered statistically significant.

### Ethics declaration

The protocol of this study was reviewed and approved by the ethics committee at the Fujian Provincial Maternal and Child Health Hospital (2014-042). All parents were informed that the data might be used for future research studies and signed written informed consent was obtained. All experiments were performed in accordance with relevant guidelines and regulations.

## Results

### Detection rates of abnormal CNVs

The included women were pregnant for 28 to 37 weeks, with an average of 29.0 ± 3.4 weeks. The ages of the participants ranged from 18 to 46 years, with an average of 32.1 ± 6.1 years. Among the 713 fetuses with ultrasound-detected abnormalities and normal karyotypes, CMA detected abnormal CNVs in 8.0% (57/713) of the cases, including pathogenic CNVs in 32 cases, VUS in 25 cases, and seven cases with benign CNVs. The detection rate of abnormal CNVs in fetuses with sonographic structural malformations (12.7%, 30/237) was significantly higher (P = 0.001) than in fetuses with non-structural abnormalities (5.7%, 27/476) (Table 1). In the case of sonographic structural malformations, the detection rates of abnormal CNVs were 31.8%, 25%, 22.2%, 17.9%, and 8.0% in fetuses with multiple malformations (7/22), central nervous system malformations (4/16), skeletal malformations (2/9), congenital anomalies of the kidney and urinary tract (7/39), and congenital heart malformations (10/125), respectively. Among fetal non-structural abnormalities, the greatest number was represented by abnormal soft indications (70.6%, 336/476), followed by FGR (25.2%, 120/476) and amniotic fluid volume abnormality and pericardial effusion (4.2%, 20/476). The detection rates of abnormal CNVs were 20%, 5.8%, and 4.8% among the fetuses with amniotic fluid volume abnormality and pericardial effusion (4/20), FGR (7/120), and abnormal soft indications (16/336), respectively (Table 2, Fig. 1).

**Table 1:** The detection rate of abnormal CNVs in 713 fetuses.

Indication for prenatal diagnosis	Number	Number of abnormal CNV	Total (%)
Sonographic structural malformation	237	30	12.7
Non-structural abnormalities	476	27	5.7

*CNV* copy number variation.

**Table 2:** Phenotypic characteristics of 713 fetuses.

Anomaly on ultrasonography	Number (% total cohort)	Number of CNV anomaly (% total anomaly)	Number of pathogenic CNV	Number of VUS CNV
Sonographic structural malformation	237 (33.2)	30 (12.7)	21	9
Multiple malformations	22 (3.1)	7 (31.8)	5	2
Central nervous disease	16 (2.2)	4 (25.0)	2	2
Skeletal malformation	9 (1.3)	2 (22.2)	2	0
Congenital anomalies of the kidney and urinary tract	39 (5.5)	7 (17.9)	4	3
Congenital heart disease	125 (17.5)	10 (8.0)	8	2
Digestive malformation	10 (1.4)	0 (0.0)	0	0
Respiratory malformation	8 (1.1)	0 (0.0)	0	0
Craniofacial malformation	8 (1.1)	0 (0.0)	0	0
Non-structural abnormalities	476 (66.8)	27 (5.7)	11	16
Amniotic fluid volume abnormality and pericardial effusion	20 (2.8)	4 (20.0)	1	3
Fetal growth restriction	120 (16.8)	7 (5.8)	5	2
Abnormal soft indication	336 (47.1)	16 (4.8)	5	11

*CNV* copy number variation, *VUS* variants of uncertain clinical significance.

### Pathogenic CNVs detected in fetuses with ultrasound abnormalities and normal karyotypes using an SNP array

Among the 32 cases with pathogenic CNVs, 24 were related to known chromosomal disorders, namely, 22q11 deletion syndrome (n = 5), 22q11.2 duplication syndrome (n = 3), 17q12 deletion syndrome (n = 3), cat eye (n = 1), 16p11.2 deletion syndrome (n = 4), Prader–Willi syndrome (n = 1), Miller–Dieker syndrome (n = 1), Wolf–Hirschhorn syndrome (n = 1), Sotos syndrome (n = 1), 7q11.23 duplication syndrome (n = 2), and 1q21.1 duplication syndrome (n = 2). Additionally, the pathogenic CNVs were associated with a loss at 17p12, 1p36.33p36.23, and 22q13.33 and a gain at 15q13.3, 1q21.1q21.2, 3q29, Xq28, and 10q11.22q11.23 (Table 3).

**Table 3:** The pathogenic copy number variation in ultrasonically abnormal fetuses.

Case	CC week	SNP array	Size (Mb)	Indication	Interpretation	Outcome
1	28^+6^	Chr22: 18,648,855–21,800,471	3.1	CHD, thymic dysplasia	Pathogenic: loss 22q11.2 (22q11deletion syndrome)	Termination of pregnancy
2	29	Chr22: 20,730,143–21,800,471	1.0	Multiple cysts of the left choroid plexus, renal cysts of the left, and varus	Pathogenic: loss 22q11.2 (22q11deletion syndrome)	Termination of pregnancy
3	33^+5^	Chr22: 18,916,842–21,800,471	2.9	VSD; Mirror-image right aortic arch	Pathogenic: loss 22q11.2 (22q11deletion syndrome)	Termination of pregnancy
4	32^+3^	Chr22: 18,648,855–21,800,471	3.1	VSD	Pathogenic: loss 22q11.2 (22q11deletion syndrome)	Termination of pregnancy
5	28^+1^	Chr22: 18,648,855–21,800,471	3.1	VSD; right aortic arch	Pathogenic: loss 22q11.2 (22q11deletion syndrome)	Termination of pregnancy
6	28^+5^	Chr22: 49,683,904–51,197,766	3.1	Echogenic bowel	Pathogenic: loss 22q13.33 (22q13 deletion syndrome)	Termination of pregnancy
7	33^+1^	Chr22: 18,649,189–21,800,471	3.1	CHD: Oval valve bulging tumor	Pathogenic: gain 22q11.2 (22q11.2 duplication syndrome), de novo	Termination of pregnancy
8	30	Chr22: 18,648,855–21,459,713	2.8	FGR	Pathogenic: gain 22q11.21 (22q11.2 duplication syndrome), inherited from mother	Normal delivery Good growth and development
9	28^+3^	Chr22: 18,648,855–21,800,471	3.1	FGR	Pathogenic: gain 22q11.21 (22q11.2 duplication syndrome), inherited from father	Cesarean section Good growth and development
10	28^+3^	Chr22: 18,888,899–18,649,190	1.7	VSD, persistent left superior vena cava	Pathogenic: gain 22q11.1q11.21 (cat eye syndrome)	Termination of pregnancy
11	28^+4^	Chr17: 34,822,465–36,404, 555	1.58	Double kidney echo enhancement	Pathogenic: loss 17q12 (17q12 deletion syndrome)	Termination of pregnancy
12	28^+^	Chr17: 34,822,465–36, 243,365	1.4	Double kidney echo enhancement	Pathogenic: loss 17q12 (17q12 deletion syndrome)	Termination of pregnancy
13	29^+4^	Chr17: 34,822,465–36,307, 773	1.48	Double kidney echo enhancement	Pathogenic: loss 17q12 (17q12 deletion syndrome)	Termination of pregnancy
14	29^+5^	Chr16: 28,810,324–29,032,280	0.22	Lateral ventricle widening, echogenic bowel, Left ventricular hyperecho	Pathogenic: loss 16p11.2 (16p11.2 deletion syndrome), de novo	Termination of pregnancy
15	31	Chr16: 29,591,326–30,176,508	0.57	Hydrocephalus	Pathogenic: loss 16p11.2 (16p11.2 deletion syndrome), de novo	Termination of pregnancy
16	28^+4^	Chr16: 29,580,020–30,190,029	0.60	Spinal dysplasia	Pathogenic: loss 16p11.2 (16p11.2 deletion syndrome), de novo	Termination of pregnancy
17	28	Chr16: 29,567,296–30,190,029	0.6	Lateral ventricle widening	Pathogenic: loss 16p11.2 (16p11.2 deletion syndrome), de novo	Termination of pregnancy
18	33^+1^	Chr15: 32,003,537–32,444,043	0.43	VSD, Aortic ride across, pulmonary stenosis,	Pathogenic: gain 15q13.3, the triple dose effect score was 1, penetrance of 5–10% in ClinGen database	Normal delivery VSD
19	37	Chr15: 31,999,631–32,444,043	0.43	Severe hydrocephalus	Pathogenic: gain 15q13.3, the triple dose effect score was 1, penetrance of 5–10% in ClinGen database	Termination of pregnancy
20	29^+6^	Chr15: 32,011,458–32,914,239	0.88	Half vertebral body	Pathogenic: gain 15q13.3, The triple dose effect score was 1, penetrance of 5–10% in ClinGen database	Termination of pregnancy
21	34	Chr1: 145,958,361–147,830,830	1.8	Lateral ventricle widening	Pathogenic: gain 1q21.1q21.2 (1q21.1 duplication syndrome)	Termination of pregnancy
22	29^+6^	Chr1: 145,995,176–147,398,268	1.4	Pulmonary stenosis; hypoplastic right heart; Tricuspid stenosis with incomplete closure	Pathogenic: gain 1q21.1q21.2 (1q21.1 duplication syndrome)	Termination of pregnancy
23	28^+4^	Chr7: 72,701,098–74,069,645	1.3	VSD, unilateral renal agenesis	Pathogenic: gain 7q11.23 (7q11.23 duplication syndrome)	Termination of pregnancy
24	32^+6^	Chr7: 72,723,370–74,143,240	1.42	FGR	Pathogenic: gain 7q11.23 (7q11.23 duplication syndrome)	Termination of pregnancy
25	29^+2^	Chr17: 525–5,204,373	5.2	Bilateral ventricle widening,Strephenopodia, cerebellum entricular dysplasia	Pathogenic: loss 17p13.3p13.2, (Miller-Dieker syndrome)	Termination of pregnancy
26	30^+4^	Chr17: 14,083,054–15,482,833	1.4	Left renal dysplasia	Pathogenic: loss 17p12, Hereditary stress susceptibility peripheral neuropathy, Inherited from mother	Termination of pregnancy
27	34^+1^	Chr4: 68,345–6,608,624	6.5	FGR, pulmonary stenosis	Pathogenic: loss 4p16.3p16.1 (Wolf-Hirschhorn syndrome)	Termination of pregnancy
28	28^+4^	Chr3: 195,743,957–197,386,180	1.6	VSD	Pathogenic: gain 3q29 (3q29 duplication syndrome)	Termination of pregnancy
29	31^+6^	Chr5: 175,416,095–177,482,506	2.0	Polyhydramnios	Pathogenic: loss 5q35.2q35.3 (Sotos syndrome)	Termination of pregnancy
30	29	ChrX; Chr1: 152,446,333–153,581,657	1.1	Bilateral ventricular walls are rough and echo is enhanced	Pathogenic: gain Xq28, 1q44, loss 1p36.33p36.23 (1p36 deletion syndrome)	Termination of pregnancy
		849,466–592,172	7.7			
		246,015,892–249,224,684	3.2			
31	33^+1^	Chr10: 46,252,072–51,903,756	5.6	FGR	Pathogenic: gain 10q11.22q11.23, reports in the DGV database, de novo	Termination of pregnancy
32	32^+6^	Chr15: 35,077,111–54,347,324	19.2	FGR	Pathogenic: uniparental disomy, Inherited from mother (Prader–Willi syndrome)	Termination of pregnancy

*CC* cordocentesis, *CHD* congenital heart disease, *VSD* ventricular septal defect, *FGR* fetal growth restriction.

### VUS detected in fetuses with ultrasound abnormalities and normal karyotypes using an SNP array

The SNP array detected that 25 fetuses carried VUS, which were associated with microdeletions, ranging from 0.22 to 5.5 Mb, and microduplications, ranging from 0.20 to 2.9 Mb (Table 4). Among the 25 cases with VUS, three fetuses were found to have 16p13.11 duplications, and three fetuses were found to have 15q11.2 deletions. The detected VUS were also associated with a gain at 17q21.31, 13q14.3, 10q24.31q24.32, 8p23.2, 4q24, 2q36.1q36.2, and 2q22.2 and a loss at 16p13.11, 1q21.1, 2q11.1.1q11.2, 3p26.3, 3p22.1, 3q28, 5p15.33p15.31, 10q11.21q11.22, 14q21.2q21.3, and Xp21.1. In addition, two fetuses lacked heterozygosity (Table 4).

**Table 4:** The variants of uncertain clinical significance in ultrasonically abnormal fetuses.

Case	CC week	SNP array	Size (Mb)	Indication	Interpretation	Outcome
1	28^+4^	Chr16: 14,897,401–16,534,031	1.6	VSD	VUS Loss 16p13.11, Hereditary stress susceptibility peripheral neuropathy, The carrier frequency in the population is less than 1%	Eutocia Good growth and development
2	28^+3^	Chr16: 15,325,072–16,272,403	0.92	Bilateral dysplasia with hydronephrosis	VUS Gain 16p13.11, The carrier frequency in the population is less than 1%, penetrance of 5–10%	Termination of pregnancy
3	32^+2^	Chr16: 15,171,146–16,309,046	1.1	Echogenic bowel	VUS: Gain 16p13.11, The carrier frequency in the population is less than 1%, penetrance of 5–10%, Inherited from mother	Eutocia Good growth and development
4	31^+1^	Chr16: 15,510,512–16,309,046	0.78	Tricuspid regurgitation	VUS Gain 16p13.11, The carrier frequency in the population is less than 1%, penetrance of 5–10%	Eutocia Good growth and development
5	33^+2^	Chr15: 22,770,421–23,277,435	0.50	VSD,Dandy-Walker malformation	VUS Loss 15q11.2, inherited from father	Termination of pregnancy
6	28	Chr15: 22,770,421–23,286,423	0.5	Echogenic bowel	VUS Loss 15q11.2, inherited from father	Cesarean growth retardation
7	29^+1^	Chr15: 22,770,421–23,082,237	0.30	Subcutaneous cyst at the back of the neck	VUS Loss 15q11.2, inherited from father	Cesarean growth retardation
8	32^+4^	Chr17: 41,774,473–42,491,805	0.70	FGR	VUS Gain 17q21.31, no report in the DGV	Cesarean growth retardation
9	28^+1^	Chr13: 52,649,105–53,172,866	0.53	Hydronephrosis, strephenopodia, tricuspid regurgitation	VUS Gain 13q14.3, no report in the DGV, de novo	Termination of pregnancy
10	33^+2^	Chr10: 102,972,457–103,179,063	0.20	Posterior fossa widened	VUS Gain 10q24.31q24.32, no report in the DGV	Eutocia Good growth and development
11	29	Chr8: 3,703,883–5,940,433	2.2	Bilateral choroid plexus cysts	VUS Gain 8p23.2, no report in the DGV database, de novo	Eutocia Good growth and development
12	29^+3^	Chr4: 106,284,925–107,545,257	1.2	VSD	VUS Gain 4q24, no report in the DGV, de novo	Loss to follow-up
13	35^+2^	Chr2: 224,459,152–225,330,583	0.85	Posterior fossa widened	VUS Gain 2q36.1q36.2, no report in the DGV, de novo	Cesarean Good growth and development
14	28^+1^	Chr2: 143,043,284–143,866,399	0.80	Pericardial effusion	VUS Gain 2q22.2, no report in the DGV, de novo	Eutocia Good growth and development
15	34	Chr1; Chr9: 145,375,770–145,770,627, 4,623,660–5,501,699	0.68, 0.86	Lateral ventricle widening	VUS Loss 1q21.1, gain 9p24.1, no report in the DGV, de novo	Eutocia Good growth and development
16	32	Chr2: 96,679,225–97,669,032	0.97	Hydronephrosis	VUS Loss 2q11.1.1q11.2, No report in the DGV database, de novo	Eutocia Good growth and development
17	33^+3^	Chr3: 1,855,754–2,663,625	0.79	Bilateral ventricle widening	VOUS Loss 3p26.3, no report in the DGV, de novo	Cesarean Good growth and development
18	29^+1^	Chr3: 42,875,130–43,309,436	0.42	Lateral ventricle widening	VUS Loss 3p22.1, no report in the DGV, de novo	Loss to follow-up
19	32^+1^	Chr3, Chr15: 188,788,120–191,331,505,23,620,191–24,978,547	2.5	Unilateral renal agenesis	VUS Loss 3q28, gain 15q11.2, no report in the DGV, de novo	Termination of pregnancy
20	29^+6^	Chr5: 4,482,234–6,636,035	2.1	Pericardial effusion	VUS Loss 5p15.33p15.31, no report in the DGV, de novo	Termination of pregnancy
21	29^+2^	Chr10: 42,433,738–48,006,310	5.5	Echogenic bowel	VUS Loss 10q11.21q11.22, The deletion fragment contains RET gene, associated with congenital megacolon, de novo	Termination of pregnancy
22	32^+4^	Chr14: 46,782,405–49,288,860	2.5	Hydrocephalus	VUS Loss 14q21.2q21.3, no report in the DGV, de novo	Eutocia Good growth and development
23	32^+6^	ChrX: 32,670,116–32,891,702	0.22	Pericardial effusion	VUS Loss Xp21.1, no report in the DGV, de novo	Loss to follow-up
24	28^+6^	Chr3, Chr5, Chr6, Chr12, Chr17, Chr21: 163,256,369–197,791,601, 41,029,137–46,313,469, 143,341,406–161,527,784, 56,011,100–77,134,151, 39,639,602–45,479,706, 28,124,165–42,352,287	99.1	Lateral ventricle widening	VUS Lack of heterozygosity 3q26.1q29, 5p13.1p11, 6q24.2q26, 12q13.2q21.2, 17q21.2q21.32, 21q21.3q22.2	Termination of pregnancy
25	32^+3^	Chr4: 133,718,289–154,569,367	20.8	FGR	VUS Lack of heterozygosity 4q28.3q31.3	Eutocia Good growth and development

*CC* cordocentesis, *VSD* ventricular septal defect, *FGR* fetal growth restriction, *VUS* variants of uncertain clinical significance.

### Benign CNVs detected in fetuses with ultrasound abnormalities and normal karyotypes using SNP array

The seven benign CNVs in fetuses were inherited from their healthy parents (Table 5). According to the Database of Genomic Variants (DGV), these genes have not been reported previously.

**Table 5:** The variants of benign in ultrasonically abnormal fetuses.

Case	CC week	SNP array	Size (Mb)	Indication	Interpretation	Outcome
1	28^+2^	Chr5: 76,983,283–77,512,158	0.5	VSD	B Gain 5q14.1, no report in the DGV, inherited from mother	Eutocia Good growth and development
2	30	Chr3 : 33,805,560–35,318,562	1.5	Pericardial effusion	B Loss 3p22.1, no report in the DGV, inherited from mother	Cesarean Good growth and development
3	31^+6^	Chr5: 154,435,034–156,727,811	2.9	Echogenic bowel	B Gain 5q33.2q33.3, no report in the DGV, inherited from father	Eutocia Good growth and development
4	28^+6^	Chr7: 139,340,641–139,769,640	0.4	Right subclavian artery vagus	B Gain7q34, no report in the DGV, inherited from mother	Eutocia Good growth and development
5	28^+6^	Chr8: 126,044,027–126,414,021	0.4	Persistent left superior vena cava, single umbilical artery	B Gain8q24.13, no report in the DGV, inherited from mother	Eutocia Good growth and development
6	29	Chr9: 122,199,202–123,921,999	1.7	Left ventricular echo, echogenic bowel	B Gain9q33.1q33.2, no report in the DGV, inherited from father	Eutocia Good growth and development
7	29	Chr9: 19,620,590–21,572,153	1.9	Left ventricular echo	B Gain18q11.2, no report in the DGV, inherited from father	Eutocia Good growth and development

*CC* cordocentesis, *VSD* ventricular septal defect, *B* benign.

### Genealogical analysis and pregnancy outcomes

The parents of nine fetuses with pathogenic CNVs were tested, and it was found that five CNVs were de nova and four were inherited. Among the 25 cases of fetuses with VUS, the parents refused genetic testing in seven cases. In the other 18 cases, the variants were confirmed to be inherited. Some pregnant women with fetuses with pathogenic CNV (n = 29) and VUS (n = 7) chose to terminate the pregnancies. However, 15 cases with VUS CNVs women chose to continue the pregnancy and showed good growth and development on postnatal examination. In the seven cases in which fetuses were found to have inherited a benign CNV from one of the normal parents, the pregnancies were continued and had a favorable outcome.

## Discussion

We retrospectively analyzed the SNP analysis data of 713 cases, and explored the correlation between phenotype characteristics of the fetuses and pathogenic CNVs. All 713 fetuses with abnormal ultrasound findings and normal karyotypes were investigated by CMA. We detected abnormal CNVs in 8.0% (57/713) of the cases, including pathogenic CNVs in 32 cases (4.5%, 32/713) and VUS in 25 cases (3.5%, 25/713). This rate of detection of abnormal CNVs was somewhat higher than that reported in previous studies^10–12^. We suggest that CMA should be considered for genetic analysis in cases with abnormal ultrasound findings in the second and third trimesters of pregnancy.

With the growth of the fetus, more fetal ultrasound abnormalities (structural and non-structural) are found during the second and third trimesters of pregnancy. In our study, the detection rate of abnormal CNVs in fetuses with sonographic structural malformations (12.7%, 30/237) was significantly higher (P = 0.001) than in fetuses with non-structural abnormalities (5.7%, 27/476). Among the fetuses with sonographic structural malformations, those with multiple malformations showed the greatest rate of CNVs (31.8%, 7/22), followed by fetuses with central nervous system malformations (25%, 4/16), congenital anomalies of the kidney and urinary tract (18.0%, 7/39), and congenital heart malformations (8%, 10/125). These findings are slightly different from those of a previous study^13^, in which cardiovascular, central nervous, gastrointestinal, and musculoskeletal system malformations were mostly associated with pathogenic CNVs.

At present, the consensus regarding the application of CMA in prenatal diagnosis does not include cases with non-structural abnormalities. There are few studies reporting the relationships between sonographic non-structural abnormalities and chromosomal duplications/deletions. In our study, among the fetuses with non-structural abnormalities, those with amniotic fluid volume abnormality and pericardial effusion showed the greatest rate of CNVs (20%, 4/20), followed by fetuses with FGR (5.8, 7/120) and abnormal soft indications (4.8%, 16/336). Compared with that reported in another study^14^, the CNV detection rate in the fetuses with sonographic non-structural abnormalities was slightly higher in this study, likely due to differences in the selected cohorts of fetuses with ultrasound anomalies in late pregnancy.

In most cases, there are direct causal relationships between pathogenic CNVs and fetal phenotypes. However, the use of genotype–phenotype associations is not always reliable in prenatal diagnosis. Our findings revealed 32 cases of pathogenic CNVs. The most common was 22q11 deletion syndrome^15^, which is associated with congenital heart disease. We also found three fetuses with 17q12 deletion syndrome^16^, which is associated with congenital anomalies of the kidney and urinary tract. The only ultrasonographic manifestations in these fetuses were double kidney echo enhancements. A CNV on human chromosome 16p11.2 is associated with autism spectrum disorders^17,18^. We detected this known 16p11.2 deletion syndrome in four fetuses, with different ultrasonographic manifestations. The ultrasonographic manifestations in three of the fetuses were hydrocephalus, spinal dysplasia, and lateral ventricle widening. The fourth fetus presented with a widened lateral ventricle, echogenic bowel, and echogenic foci within the left ventricle. Owing to the limitations of ultrasound, fetal phenotypes are often incomplete, which may complicate prenatal counseling^19^. Therefore, the application of genomic detection techniques is important in fetuses with ultrasound abnormalities.

We detected 25 fetuses with VUS, of which three fetuses had 16p13.11 duplications and one had a 16p13.11 deletion. According to the literature^20–23^, gain and loss at 16p13.11 have been associated with autism, schizophrenia, and epilepsy. The carrier frequencies of duplications and deletions at the 16p13.11 locus are less than 1% in the general population, but the penetrance is 5–10%^24^; therefore, we defined the CNVs at 16p13.11 as VUS. We also detected 15q11.2 deletions in three fetuses. The ultrasonographic manifestations in these fetuses included ventricular septal defect (VSD), a Dandy–Walker malformation, an echogenic bowel, and a subcutaneous cyst at the back of the neck. Genetic analysis showed that these deletions were inherited from one of the normal parents. However, there are some reports of 15q11.2 deletions causing neurodevelopmental alterations^25,26^. Thus, we also defined the 15q11.2 deletions as VUS. Another 15 mutations were defined as VUS because these CNVs have not been reported in the DGV and were identified as de novo variants by pedigree analysis. Two cases were identified to lack heterozygosity. The parents of these two cases refused to undergo a pedigree test, and therefore, we defined the lack of heterozygosity as VUS. Seven fetuses were found to have inherited their CNVs from their normal parents. As there are no reports of these CNVs in the DGV, they were defined as benign CNVs. At present, the greatest difficulty in the use of SNP-array results in prenatal diagnosis lies in the correct interpretation of VUS. Literature reports show that the rates of VUS detection are 1.4–12.3%^11,27,28^; hence, the rate found in our study (3.5%) was consistent with previous findings.

Following the application of an SNP array to rule out genomic abnormalities, pregnant women generally choose to deliver their babies, thus avoiding unnecessary termination of pregnancy. This outcome emphasizes the importance of CMA in genetic counseling. In our study, 29 cases of fetal sonographic structural malformations with pathogenic CNVs resulted in pregnancy termination; however, in three cases, the parents chose to deliver and neonates had good outcomes no other abnormalities were found except one with VSD. Of the 25 cases of fetuses with VUS, in 15 cases, the parents chose to deliver, and the babies showed good growth and development after birth. We also found that seven cases associated with benign CNVs continued their pregnancies and had a favorable outcome. According to our experience, if the observations by ultrasound associated with chromosomal anomalies and other fatal malformation are excluded, the pregnant women may have increased confidence in continuing pregnancy, especially CNVs were inherited.

There were some limitations to our study. First, we only used the SNP array to detect CNVs in fetuses with ultrasound-detected abnormalities and normal karyotyping analysis results. Thus, we cannot rule out single-gene mutations. In recent years, next-generation sequencing has been used as a new technique for genetic testing to detect single-gene mutations and CNVs. This technology may provide a more comprehensive prenatal genetic diagnosis for fetuses with ultrasound-detected abnormalities and a better assessment of the fetal prognosis. Second, the origins of some VUS were not traced, and thus, the sample size should be increased to further study these VUS. In addition, the numbers of cases in the subsystems of some ultrasound abnormalities were insufficient, and therefore, it was impossible to group those cases at a deeper level. In the future, more cases need to be analyzed, or multicenter collaborations are required, to improve the statistical reliability of the findings.

## Conclusion

CMA can be used as an effective method for genetic diagnosis of ultrasonically abnormal late pregnancy fetuses. The detection rate of CMA was different for different types of ultrasonically abnormal late pregnancy fetuses. CMA can improve the detection rate of chromosomal abnormalities that cannot be detected by karyotype analysis. In genetic counselling, CMA should be utilized based on the type of ultrasonic abnormality observed.

## Data Availability

All data generated during and/or analyzed during the current study are available upon request by contact the corresponding author.
